# Association of Toll-Like Receptor 3 Single-Nucleotide Polymorphisms and Hepatitis C Virus Infection

**DOI:** 10.1155/2017/1590653

**Published:** 2017-01-03

**Authors:** Mashael R. Al-Anazi, Sabine Matou-Nasri, Ayman A. Abdo, Faisal M. Sanai, Saad Alkahtani, Saud Alarifi, Abdullah A. Alkahtane, Hamad Al-Yahya, Daoud Ali, Mohammed S. Alessia, Bushra Alshahrani, Mohammed N. Al-Ahdal, Ahmed A. Al-Qahtani

**Affiliations:** ^1^Department of Infection and Immunity, Research Center, King Faisal Specialist Hospital & Research Center, Riyadh, Saudi Arabia; ^2^Cell and Gene Therapy Group, Medical Genomics Research Department, King Abdullah International Medical Research Center, Ministry of National Guard, Riyadh, Saudi Arabia; ^3^Section of Gastroenterology, Department of Medicine, College of Medicine, King Saud University, Riyadh, Saudi Arabia; ^4^Liver Disease Research Center, King Saud University, Riyadh, Saudi Arabia; ^5^Gastroenterology Unit, Department of Medicine, King Abdulaziz Medical City, Jeddah, Saudi Arabia; ^6^Department of Zoology, Science College, King Saud University, Riyadh, Saudi Arabia; ^7^Department of Biology, Science College, AI-Imam Muhammad Ibn Saud Islamic University, Riyadh, Saudi Arabia; ^8^Department of Microbiology and Immunology, Alfaisal University School of Medicine, Riyadh, Saudi Arabia

## Abstract

Toll-like receptor 3 (TLR3) plays a key role in innate immunity by recognizing pathogenic, double-stranded RNAs. Thus, activation of TLR3 is a major factor in antiviral defense and tumor eradication. Although downregulation of* TLR3* gene expression has been mainly reported in patients infected with hepatitis C virus (HCV), the influence of TLR3 genotype on the risk of HCV infection, HCV-related cirrhosis, and/or hepatocellular carcinoma (HCC) remains to be determined. Single-nucleotide polymorphisms (SNPs) within the* TLR3 *gene and their associations with HCV-related disease risk were investigated in a Saudi Arabian population in this study. Eight* TLR3 *SNPs were analyzed in 563 patients with HCV, which consisted of 437 patients with chronic HCV infections, 88 with HCV-induced liver cirrhosis, and 38 with HCC. A total of 599 healthy control subjects were recruited to the study. Among the eight* TLR3 *SNPs studied, the rs78726532 SNP was strongly associated with HCV infection when compared to that in healthy control subjects. The rs5743314 was also strongly associated with HCV-related liver disease progression (cirrhosis and HCC). In summary, these results indicate that distinct genetic variants of* TLR3 *SNPs are associated with HCV infection and HCV-mediated liver disease progression in the Saudi Arabian population.

## 1. Introduction

Hepatitis C virus (HCV) is an enveloped virus containing a positive-sense, single-stranded RNA genome that infects hepatocytes specifically through a noncytopathic process. It is a member of the genus* Hepacivirus* and belongs to the Flaviviridae family. HCV is a blood-borne pathogen and results in a significant public health problem, affecting approximately 180 million people globally [1, 2]. Owing to its ability to escape the host's defense mechanisms, HCV infection is considered persistent. Nearly 80% of all infected individuals develop chronic infection that persists for many years. Chronically infected individuals develop liver complications that include fibrosis and cirrhosis. Once cirrhosis has occurred, 3–5% of these individuals will develop hepatocellular carcinoma (HCC) [2, 3].

HCV-related diseases are treated with interferon-alpha (IFN-*α*) and ribavirin (RBV), antiviral agents that are used as the standard of care for patients with chronic HCV infection. However, this treatment regimen is effective in only 50–60% of patients with HCV [4]. Recently, there has been a major advancement in the treatment and management of HCV through the development of direct-acting anti-HCV drugs in addition to drug-induced activation of the host's cell-mediated immunity. Toll-like receptors (TLRs), a family of evolutionary conserved receptors that recognizes pathogens, have emerged as key regulators of both innate and adaptive immune responses. Lately, activation of immune cells through TLR agonists, which triggers interferon production, is being developed for therapy against HCV infection [5].

TLRs are a group of molecules that are essential for the innate immune response against infecting pathogens [6]. The effect of TLRs is mediated by sensing of molecules called pathogen-associated microbial patterns (PAMPs) [7]. In humans, there are 10 TLRs, which are expressed in different organs of the immune system [6]. In particular, TLR3 is encoded by a gene located on 4q35.1 and spans five exons. It is expressed intracellularly and recognizes double-stranded RNA (dsRNA) by responding with subsequent increases in interferon-*α* transcription, and its cellular effect is mediated through the recruitment of downstream signaling molecules such as retinoic acid-inducible gene 1 (RIG-I), melanoma differentiation-associated protein 5 (MDA5), and NACHTLRR-PYD-containing protein-3 (NALP3) [8]. Lately, TLR3-mediated antitumor activities inhibiting HCC development and progression have also been described [9–11].

Several studies have shown that genetic variations in the TLR3 gene are associated with susceptibility and/or resistance to numerous infectious and immune diseases [12], including acquired immune deficiency syndrome (AIDS) [13, 14], hepatitis B viral (HBV) infection [15], liver diseases in HCV-infected patients [16], predisposition to tick-borne encephalitis [17], human herpes simplex virus type 2 (HHV-2) infection [18], cutaneous candidiasis [19], autoimmunity [19], and type 1 diabetes mellitus [20].

In this study, we aimed to investigate the influence of* TLR3 *single-nucleotide polymorphisms (SNPs) on the susceptibility to HCV infection and HCV-related cirrhosis with or without HCC in a Saudi Arabian population.

## 2. Patients and Methods

### 2.1. Patients

The study protocol conformed to the 1975 Declaration of Helsinki and was approved by the Institutional Review Boards of King Khalid University Hospital (KKUH), Prince Sultan Military Medical City (PSMMC), and King Faisal Specialist Hospital and Research Center (KFSHRC). This study included 563 patients with HCV and 599 healthy control subjects. Participants in this study were recruited from the three tertiary care hospitals mentioned above in Riyadh, Saudi Arabia. The patients were divided into three groups: group 1 consisted of patients with chronic HCV (*n* = 437), group 2 consisted of patients with liver cirrhosis (LC) (*n* = 88), and group 3 consisted of patients with HCC (*n* = 38). Chronic HCV was diagnosed by the detection of anti-HCV antibodies and persistent presence of serum HCV RNA for more than 6 months, without any signs of liver complications. LC was diagnosed clinically by the detection of ascites, esophageal varices, and imaging findings on ultrasonography, transient elastography, computed tomography (CT), and magnetic resonance imaging (MRI) [21, 22]. HCC was confirmed on the basis of a pathological examination and/or elevation of blood alpha-fetoprotein (>400 ng/mL) in conjunction with CT, MRI, or ultrasonography scans.

Blood samples from healthy control individuals were obtained from blood donors in the participating hospitals and were HBs antigen (HBsAg) and HBe antigen (HBeAg) negative, while also lacking any serological markers for HCV, HBV, and HIV. Informed consent was obtained from all participants prior to enrollment in the study, and their demographic and clinical data were also recorded.

### 2.2. DNA Extraction and TLR3 SNP Genotyping

DNA was purified from blood using Gentra Puregene Blood kit (Qiagen, Hilden, Germany) according to the manufacturer's instructions. All samples were genotyped for the eight* TLR3* SNPs via a PCR-based genotyping assay with two sets of specific primers designed using Primer3 v.0.4.0 (http://frodo.wi.mit.edu/primer3/) for* TLR3* amplification: set 1 consisted of 5′-GCTGGAAAATCTCCAAGAGC-3′ and 5′-AGAGACCAAGCCAGCTAACC-3′; and set 2 consisted of 5′-GCGCTAAAAAGTGAAGAACTGG-3′ and 5′-GGGCTCTTGACCATCGTACT-3′. PCR reactions were performed on the Veriti 96-well thermal cycler (Applied Biosystems, California, USA) under the following conditions: 5 min initial denaturation at 95°C, followed by 40 cycles of 95°C for 1 min, 58°C for 45 s, 72°C for 1 min, and a 5 min final extension at 72°C. The amplified PCR products were analyzed by direct sequencing using the BigDye® Terminator v3.1 Cycle Sequencing Kit according to the manufacturer's instructions (BigDye Terminator v3.1 Cycle Sequencing Kit, Applera, Connecticut, USA). Sequencing products were purified using DyeEx spin column and were analyzed on the ABI 3700 DNA Analyzer (Applied Biosystems, California, USA).

### 2.3. Statistical Analysis

The genotypic and allelic distribution of TLR3 SNPs between the patients and healthy control groups were evaluated by Pearson's *χ* ^2^ test. The association between the SNPs and the disease groups was calculated under dominant and recessive genetic models and was expressed in terms of an odds ratio (OR) and their 95% confidence intervals (CI). A *p* value of less than 0.05 was considered statistically significant. Statistical analysis was performed using SPSS version 20.0 (SPSS Inc., Chicago, IL, USA). The SNPs were tested for Hardy–Weinberg equilibrium (HWE) and a *p* value of 0.01 was set for the analysis.

## 3. Results

### 3.1. Genotype and Allele Frequency Distributions of the TLR3 Polymorphisms Associated with HCV Infection

Polymorphisms of TLR3 gene were previously described as genetic factors associated with the susceptibility to HBV infection in a Saudi Arabian population [15]. Here, we analyzed the allelic frequency distribution of eight* TLR3* SNPs in patients with HCV (*n* = 563) in comparison with that in healthy control subjects (*n* = 599). The baseline and clinical characteristics of the study subjects, HCV-infected patients, and healthy control subjects are presented in Table 1. Older age and gender were significantly linked to a higher risk for chronic HCV infection. Predictive indicators of the progression of liver disease such as body mass index (BMI), platelet count, and HCV viral load (ALT, ASP, and ALP) were also assessed in patients with HCV. End-stage liver disease was significantly correlated with a reduction in platelet count and increase in AST levels, whereas no significant differences were observed among other groups.

Genotype frequencies and distributions of each* TLR3* SNPs were in accordance with HWE in both the control and HCV-infected groups (Table 2).

We also confirmed the relevance of the* TLR3* SNPs as markers for chronic HCV infection in patients compared to that in the noninfected healthy control subjects. The genotype distribution and allele frequency for TLR3 polymorphisms between patients with HCV and the control group are summarized in Table 3. Our results showed that the GG genotype of TLR3 rs78726532 was associated with the susceptibility to HCV infection when compared with healthy controls (OR = 0.264, 95% CI = 0.074–0.942, and *p* value = 0.027). No significant difference in the genotype and allele distributions of rs5743311, rs5743312, and rs1879026; rs5743313 and rs5743314; and rs5743315 and rs111611328* TLR3* SNPs was observed in patients with HCV when compared with that in healthy controls.

### 3.2. Genotype and Allele Frequency Distributions of the TLR3 Polymorphisms Associated with HCV-Related Liver Diseases

Genotype and allele distributions were also determined in patients with HCV that were developing cirrhosis, a late-stage liver disease marked by inflammation. Among the eight TLR3 polymorphisms, only two SNPs (rs5743313 and rs5743314) showed a significant association with the risk for cirrhosis in patients with HCV when compared with patients with chronic HCV infections (Table 4). The rs5743313 SNP was dominantly associated with liver cirrhosis (OR = 1.605, CI = 1.009–2.554, and *p* value = 0.045). In addition, the rs5743314 GC genotype was significantly correlated with the risk for cirrhosis (OR = 1.702, 95% CI = 1.050–2.759, and *p* value = 0.029) when compared with HCV patients (Table 4). The percentage of the rs5743314 C allele among the patients diagnosed with liver cirrhosis (34.6%) was higher than that of patients with chronic HCV (OR = 1.468, 95% CI = 1.040–2.072, and *p* value = 0.03), and the C allele was found to be dominantly associated (OR = 1.730, 95% CI = 1.085–2.757, and *p* value = 0.0204), suggesting that the C allele may contribute to HCV progression. No significant difference in the genotype and allele distributions of the other SNPs was observed between patients with HCV-related liver cirrhosis and those with chronic HCV (Table 4).

To assess the influence of TLR3 polymorphisms on the risk of progression of end-stage liver diseases (liver cirrhosis and HCC), the genotype and allelic distributions were analyzed between patients with chronic HCV and patients diagnosed with liver cirrhosis and developing HCC. The* TLR3* SNP rs5743314 that was previously described to be associated with risk for cirrhosis was also found to be associated with the risk for chronic HCV-related end-stage liver disease progression (rs5743314 GC genotype: OR = 1.545, 95% CI = 1.022–2.334, and *p* value = 0.0383), also the dominant model of rs5743314 associated with HCV liver disease progression (OR = 1.523, 95% CI = 1.021–2.271, and *p* value = 0.0385) (Table 5). The CT genotype and the dominant model TT+CT versus CC of rs5743313 were only slightly statistically significantly associated with liver cirrhosis and HCC (*p* value = 0.054 and 0.053, resp.). Similarly, the C allele and the recessive model CC.VS.GC+GG of rs111611328 were slightly statistically associated when compared to those in patients with chronic HCV with cirrhotic and HCC individuals (OR = 4.683, 95% CI = 1.041–21.062, and *p* value = 0.088; OR = 0.096, 95% CI = 0.004–2.362, and *p* value = 0.062; resp.) (Table 5). No significant difference in the genotype and allele distributions of the other SNPs was observed between patients with chronic HCV and those with HCV diagnosed with liver cirrhosis and HCC.

### 3.3. Haplotype Analyses

**Figure 1:**
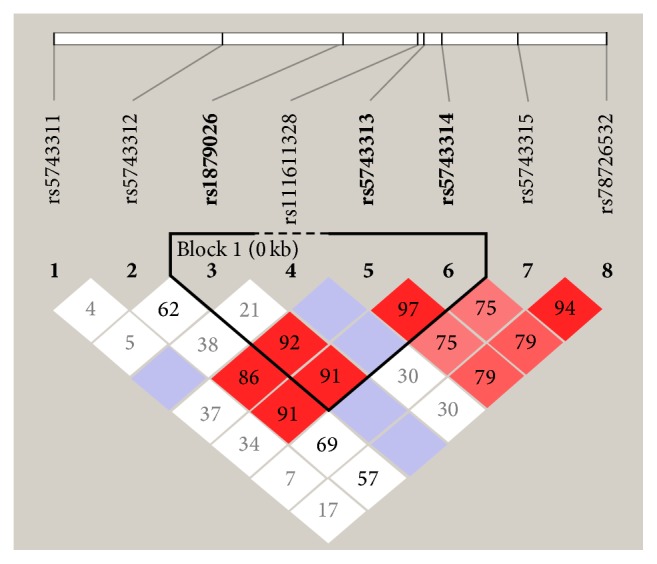
LD plot of SNPs in the* TLR3* gene region. The plot was generated by Haploview 4.2. Numbers in squares show LD between the SNPs.

The haplotype combinations for three* TLR3* SNPs (rs1879026, rs5743313, and rs5743314) and their genotypic distribution within the two comparative studies (patients with HCV diagnosed with cirrhosis compared to patients with chronic HCV) were established to determine the linkage disequilibrium (LD) pattern and the frequency of the haplotypes. The frequency of the haplotype GTC (freq. = 0.27), which includes the risk allele C for rs5743314, was statistically associated with the risk of progression to end-stage HCV-related liver diseases (*p* value = 0.0248), despite the absence of any correlation with the two other* TLR3* SNPs (rs1879026 and rs5743313) with risk for HCV infection or end-stage liver disease (Table 6). Similarly, haplotype analysis was performed between patients with chronic HCV and patients diagnosed with liver cirrhosis and HCC, revealing that one out of three haplotypes was close to becoming significant, which includes the three* TLR3* SNPs rs1879026-G, rs5743313-T, and rs5743314-C, with a *p* value = 0.0632 (Table 7, Figure 1).

## 4. Discussion

Over the past 25 years, HCV has been a major causative factor in parenteral-acquired hepatitis and has received considerable attention owing to its ability to evade the host's defense mechanisms and a lack of a protective vaccine [23]. HCV persistence leads to chronic and progressive end-stage liver diseases, including HCC, which often necessitates liver transplantations for the patient, thus making HCV infections an important disease burden. Therefore, there is an urgent need for predictive genetic tools such as those based on SNP profiling (referred as personalized medicine), to characterize patients with susceptibility to HCV infection and HCV-mediated liver diseases. This will allow for better predictions in HCV infection progression rates and improvements in the outcome of standard therapies involving pegylated IFN-*α* combined with ribavirin [24]. TLR3, a sensor of the host immune system activated by viral dsRNA and responsible for the production of IFN, has been demonstrated to play an essential role during HCV infection and clearance, as well as in the severity of liver diseases. Thus, SNP genotyping of the* TLR3* gene might be a good candidate approach to predicting the progression of HCV infections. Here, we investigated the influence of genetic variants within* TLR3* and determined the degree of association with HCV infection and HCV-related liver damage that results in cirrhosis and HCC. Among the eight* TLR3* SNPs genotyped, rs78726532 was strongly associated with HCV infection when compared to healthy control subjects. A protective role of the rs78726532 GG genotype for HCV infection was also observed. Three other* TLR3* SNPs (rs5743313, rs5743314, and rs111611328) were also associated with HCV-related end-stage liver disease progression (liver cirrhosis and HCC).

Initially, low levels of* TLR3* gene expression were detected in various liver cells, including nonparenchymal cells (i.e., Kupffer cells, sinusoidal endothelial cells, dendritic cells, stellate cells, and biliary epithelial cells) and hepatic lymphocytes [25–28]. TLR3 is mainly located on the endosome-lysosome membrane but can also be expressed on the plasma membrane of some cells. The host's immune defense is initiated with the presence of a byproduct of the replicative cycle of many single-stranded RNA viruses that could interact with the dsRNA-sensing receptor, TLR3. TLR3 activation results in the stimulation of interferon-regulatory factor- (IRF-3-) dependent type I IFN responses, NF-kB-dependent proinflammatory cytokine production, and stimulation of IFN genes (ISGs) that suppress HCV replication [29]. During early stages of HCV infection, TLR3 expression is upregulated by IFN, indicating a positive feedback regulation. Thus, TLR3 signaling may have pleiotropic functions and is involved in inflammation leading to HCV clearance during the course of acute liver injury. Herein, a protective role of the* TLR3* rs78726532 GG genotype for HCV infection was observed, indicating a robust expression and function of the TLR3 receptor, which is an important determinant for viral clearance [30]. In a previous study assessing the function of TLR3 in macrophages of patients with chronic HCV, the intronic* TLR3* rs1316816 SNP was found to be associated with high TLR3 expression and HCV clearance [31]. In our study, monitoring the level of expression of TLR3 in patients harboring the* TLR3* rs78726532 SNP would be of interest.

Among the eight* TLR3* SNPs genotyped, rs78726532 was strongly associated with HCV infection when compared to that in their healthy counterparts. Multiple SNPs positioned in the* TLR3* gene targeted to assess the risk of HCV infection have yielded inconsistent results. Indeed, Sá et al., 2015, demonstrated that the rs5743305 and rs3775291 SNPs were not associated with a risk for HCV infection [32]. This was inconsistent with a study by Medhi et al., 2011, that analyzed polymorphisms at the promoter region of the TLR3 gene [33], as well as a recent meta-analysis that concluded* TLR3* gene polymorphisms (mainly rs3775291) were associated with a risk for HCV infection [34]. This inconsistency may be explained by the involvement of genetic factors due to racial and ethnic differences, as well as controversies in regard to whether TLR3 expression is upregulated [35, 36] or downregulated [37, 38] in peripheral blood mononuclear cells from patients with chronic HCV. Furthermore, a growing body of evidence suggests that TLR3 signaling is inhibited or diminished during HCV infection, a mechanism that may contribute to HCV modulation of the host immune system leading to chronic infection. In addition, it has been shown that HCV can modulate p53 function, which has been demonstrated to activate* TLR3* transcription by binding to the p53 consensus site in the* TLR3* promoter [39]. A deeper understanding of the molecular and regulatory system that results in TLR3 downregulation is crucial to an improvement of the TLR3-mediated innate immunity as a therapeutic approach.

In this present study, we showed that three other* TLR3* SNPs (rs5743313, rs5743314, and rs111611328) were strongly associated with HCV-related end-stage liver disease progression (liver cirrhosis and HCC). However, several studies have suggested that TLR3 may contribute to resistance against HCV infection, even though TLR3 appears to have no role in disease advancement after a chronic infection is established [16, 40]. TLR3 polymorphisms (rs1879026 and rs3775290) have been described to be associated with a risk for HBV-related liver diseases in a Chinese population [41], whereas the same nine SNPs of our study were not correlated with any susceptibility to HBV-related liver diseases in the Saudi Arabian population [15]. Therefore, the impact of TLR3 polymorphisms may act in an ethnic- and viral-specific manner. At the cellular and molecular level, there is a growing body of evidence indicating that TLR3 plays a role in cirrhosis pathogenesis and hepatocarcinogenesis [42, 43]. For example, it has been documented that dsRNA activates TLR3 which subsequently results in NK cell accumulation and activation leading to liver inflammation. Such process could contribute to cirrhosis and HCC if left untreated [44–46]. Previous studies using a rat model showed that activated TLR3 could inhibit HCC development and progression by inhibiting cell invasion and inducing apoptosis in cancer cells [9, 10]. Therefore, the strong association of three* TLR3* SNPs (rs5743313, rs5743314, and rs111611328) with HCV-related end-stage liver disease progression (cirrhosis and HCC) indicates an impairment of TLR3 function in the prevention of excessive inflammation and control of liver regeneration.

## 5. Conclusion

In conclusion, we showed that distinct TLR3 polymorphisms were associated with susceptibility to HCV infection and to HCV-related end-stage liver disease progression. These findings may act as indicators for differential TLR3 structures distinctively involved in immunity and carcinogenesis. A better understanding of these distinguishable structural and functional features will be helpful for developing therapeutic applications against TLR3-mediated HCV infections and TLR3-mediated, HCV-related liver diseases.

## Figures and Tables

**Table 1:** Baseline and clinical characteristics of HCV-infected patients and healthy control subjects.

Variable	Chronic HCV (*n* = 437)	Liver cirrhosis (*n* = 88)	HCC (*n* = 38)	Healthy control (599)	*p* ^a^ value
Age (yrs.)	43.23 ± 22.25	53.20 ± 19.79	63.23 ± 10.70	30.79 ± 8.90	<0.0001
Sex					
Male count (%)	214 (49%)	44 (50%)	18 (47.4%)	577 (96.3%)	<0.0001
Female count (%)	223 (51%)	44 (50%)	20 (52.6%)	22 (3.7%)	
BMI^*∗*	29.2 (25.67–33.39)	30.06 (26.22–33.06)	25.99 (21.89–31.39)		0.804
Platelet (per 10^9^/L)^*∗*^	230 (178.75–282.25)	163.5 (108.50–205.50)	104.00 (51.50–139.00)		<0.0001
ALT^*∗∗*^	87.58 ± 119.76	93.18 ± 68.88	114.33 ± 31.78		0.738
AST^*∗∗*^	55.36 ± 41.87	77.28 ± 47.20	91.33 ± 24.58		<0.0001
ALP^*∗∗*^	113.37 ± 78.91	133.66 ± 90.04	147.00 ± 96.70		0.072
HCV viral Load (log⁡10)^*∗*^	6.04 (5.42–6.53)	6.08 (5.57–6.45)	5.98 (2.79–7.24)		0.552

^*∗*^Values are expressed as median interquartile range (25th–75th). ^*∗∗*^Values are expressed as mean ± SD.

*p* ^a^: nonparametric test and one-way ANOVA for continuous data and Chi square test for categorical data.

ALT: alanine aminotransferase; AST: aspartate aminotransferase; ALP: alkaline phosphatase.

**Table 2:** Heterozygosity, HWE, and minor allele frequency of the TLR3 SNPs.

Name	Position	ObsHET	PredHET	HWpval	MAF	Alleles
rs5743311	187000164	0.036	0.035	1	0.018	G:A
rs5743312	187000256	0.255	0.267	0.3738	0.159	C:T
rs1879026	187000321	0.295	0.288	0.7829	0.175	G:T
rs111611328	187000364	0.006	0.01	0.021	0.005	G:C
rs5743313	187000367	0.417	0.394	0.2523	0.269	C:T
rs5743314	187000375	0.408	0.39	0.3682	0.265	G:C
rs5743315	187000416	0.211	0.198	0.2656	0.112	C:A
rs78726532	187000464	0.215	0.202	0.2266	0.114	A:G

**Table 3:** Genotypic distribution of TLR3 gene polymorphisms when all HCV-infected patients were compared to healthy control group.

SNPs	Genotype/allele distribution	Healthy controls	HCV patients	OR (95% CI)	*χ* ^2^	*p* value
		*n* = 599	*n* = 563			
rs5743311	AA	1 (0.17%)	0 (0%)	0.360 (0.015–8.846)	0.93	0.336
	AG	10 (1.67%)	18 (3.2%)	1.942 (0.889–4.244)	2.87	0.091
	GG	588 (98.16%)	545 (96.8%)	Ref		
	**A**	12 (1%)	18 (1.6%)	1.606 (0.770–3.348)	1.62	0.203
	G	1186 (99%)	1108 (98.4%)			
	AA+AG.VS.GG			1.765 (0.826–3.772)	2.21	0.137
	AA.VS.AG+GG			2.825 (0.115–69.479)	0.94	0.332

rs5743312	TT	18 (3.01%)	16 (2.84%)	0.959 (0.482–1.905)	0.01	0.903
	CT	141 (23.54%)	139 (24.69%)	1.063 (0.811–1.393)	0.2	0.657
	CC	440 (73.46%)	408 (72.47%)	Ref		
	**T**	177 (14.77%)	171 (15.19%)	1.033 (0.822–1.297)	0.08	0.781
	C	1021 (85.23%)	955 (84.81%)			
	TT+CT.VS.CC			1.051 (0.811–1.362)	0.14	0.705
	TT.VS.CT+CC			1.059 (0.535–2.098)	0.03	0.869

rs1879026	TT	23 (3.84%)	15 (2.66%)	0.701 (0.361–1.363)	1.11	0.292
	GT	160 (26.71%)	161 (28.6%)	1.082 (0.835–1.401)	0.35	0.552
	GG	416 (69.45%)	387 (68.74%)	Ref		
	**T**	206 (17.2%)	191 (16.96%)	0.984 (0.792–1.221)	0.02	0.882
	G	992 (82.8%)	935 (83.04%)			
	TT+GT.VS.GG			1.034 (0.806–1.326)	0.07	0.793
	TT.VS.GT+GG			1.459 (0.753–2.825)	1.27	0.2602

rs5743313	TT	42 (7.01%)	37 (6.57%)	0.952 (0.595–1.524)	0.04	0.838
	CT	250 (41.74%)	242 (42.98%)	1.046 (0.824–1.329)	0.14	0.71
	CC	307 (51.25%)	284 (50.44%)	Ref		
	**T**	334 (27.88%)	316 (28.06%)	1.009 (0.842–1.210)	0.01	0.921
	C	864 (72.12%)	810 (71.94%)			
	TT+CT.VS.CC			1.033 (0.821–1.300)	0.08	0.783
	TT.VS.CT+CC			1.072 (0.678–1.694)	0.09	0.766

rs5743314	CC	44 (7.35%)	37 (6.57%)	0.897 (0.563–1.429)	0.21	0.646
	GC	249 (41.57%)	239 (42.45%)	1.023 (0.805–1.301)	0.04	0.8501
	GG	306 (51.09%)	287 (50.98%)	Ref		
	**C**	337 (28.13%)	313 (27.8%)	0.984 (0.821–1.179)	0.03	0.858
	G	861 (71.87%)	813 (72.2%)			
	CC+GC.VS.GG			1.004 (0.798–1.264)	0	0.971
	CC.VS.GC+GG			1.127 (0.716–1.773)	0.27	0.605

rs5743315	AA	9 (1.5%)	3 (0.53%)	0.352 (0.095–1.309)	2.65	0.104
	AC	123 (20.53%)	118 (20.96%)	1.014 (0.763–1.347)	0.01	0.926
	CC	467 (77.96%)	442 (78.51%)	Ref		
	**A**	141 (11.77%)	124 (11.01%)	0.928 (0.718–1.199)	0.33	0.566
	C	1057 (88.23%)	1002 (88.99%)			
	AA+AC.VS.CC			0.969 (0.733–1.280)	0.05	0.822
	AA.VS.AC+CC			2.847 (0.767–10.572)	2.67	0.102

rs111611328	CC	3 (0.5%)	1 (0.18%)	0.353 (0.037–3.404)	0.89	0.346
	GC	6 (1%)	5 (0.89%)	0.883 (0.268–2.909)	0.04	0.837
	GG	590 (98.5%)	557 (98.93%)	Ref		
	**C**	12 (1%)	7 (0.62%)	0.618 (0.243–1.576)	1.03	0.309
	G	1186 (99%)	1119 (99.38%)			
	CC+GC.VS.GG			0.706 (0.250–1.997)	0.43	0.509
	CC.VS.GC+GG			2.829 (0.293–27.275)	0.88	0.347

rs78726532	GG	12 (2%)	3 (0.53%)	0.264 (0.074–0.942)	4.84	0.027
	AG	120 (20.03%)	118 (20.96%)	1.039 (0.781–1.382)	0.07	0.793
	AA	467 (77.96%)	442 (78.51%)	Ref		
	**G**	144 (12.02%)	124 (11.01%)	0.906 (0.702–1.169)	0.58	0.447
	A	1054 (87.98%)	1002 (88.99%)			
	GG+AG.VS.AA			0.969 (0.733–1.280)	0.05	0.822
	GG.VS.AG+AA			3.816 (1.071–13.594)	4.92	0.026

Note: risk alleles are marked in bold letters.

**Table 4:** Genotypic distribution of TLR3 gene polymorphisms when chronic HCV patients were compared to patients diagnosed with liver cirrhosis.

SNPs	Genotype/allele distribution	Chronic HCV	Liver cirrhosis	OR (95% C.I.)	*χ* ^2^	*p* value
		*n* = 437	*n* = 88			
rs5743311	AA	0 (0%)	0 (0%)	4.829 (0.095–244.994)	NaN	1.00
	AG	15 (3.43%)	1 (1.14%)	0.323 (0.042–2.480)	1.31	0.253
	GG	422 (96.57%)	87 (98.86%)	Ref		
	**A**	15 (1.72%)	1 (0.57%)	0.327 (0.043–2.494)	1.29	0.515
	G	859 (98.28%)	175 (99.43%)			
	AA+AG.VS.GG			0.323 (0.042–2.480)	1.31	0.253
	AA.VS.AG+GG			0.202 (0.004–10.263)	NaN	1.00

rs5743312	TT	15 (3.43%)	1 (1.14%)	0.301 (0.039–2.321)	1.49	0.223
	CT	110 (25.17%)	18 (20.45%)	0.740 (0.422–1.299)	1.11	0.292
	CC	312 (71.4%)	69 (78.41%)	Ref		
	**T**	140 (16.02%)	20 (11.36%)	0.672 (0.408–1.108)	2.46	0.117
	C	734 (83.98%)	156 (88.64%)			
	TT+CT.VS.CC			0.687 (0.397–1.190)	1.81	0.178
	TT.VS.CT+CC			3.092 (0.403–23.720)	1.31	0.253

rs1879026	TT	13 (2.97%)	2 (2.27%)	0.698 (0.154–3.169)	0.22	0.639
	GT	129 (29.52%)	21 (23.86%)	0.739 (0.433–1.260)	1.24	0.265
	GG	295 (67.51%)	65 (73.86%)	Ref		
	**T**	155 (17.73%)	25 (14.2%)	0.768 (0.486–1.213)	1.29	0.257
	G	719 (82.27%)	151 (85.8%)			
	TT+GT.VS.GG			0.735 (0.439–1.231)	1.37	0.241
	TT.VS.GT+GG			1.318 (0.292–5.948)	0.13	0.718

rs5743313	TT	28 (6.41%)	8 (9.09%)	1.825 (0.772–4.317)	1.92	0.165
	CT	179 (40.96%)	44 (50%)	1.570 (0.970–2.543)	3.40	0.065
	CC	230 (52.63%)	36 (40.91%)	Ref		
	**T**	235 (26.89%)	60 (34.09%)	1.406 (0.995–1.987)	3.76	0.052
	C	639 (73.11%)	116 (65.91%)			
	TT+CT.VS.CC			1.605 (1.009–2.554)	4.03	0.045
	TT.VS.CT+CC			0.685 (0.301–1.557)	0.83	0.363

rs5743314	CC	28 (6.41%)	8 (9.09%)	1.902 (0.803–4.505)	2.19	0.138
	GC	176 (40.27%)	45 (51.14%)	1.702 (1.050–2.759)	4.72	0.029
	GG	233 (53.32%)	35 (39.77%)	Ref		
	**C**	232 (26.54%)	61 (34.66%)	1.468 (1.040–2.072)	4.79	0.03
	G	642 (73.46%)	115 (65.34%)			
	CC+GC.VS.GG			1.730 (1.085–2.757)	5.38	0.0204
	CC.VS.GC+GG			0.685 (0.301–1.557)	0.83	0.363

rs5743315	AA	3 (0.69%)	0 (0%)	0.694 (0.035–13.585)	0.61	0.434
	AC	92 (21.05%)	18 (20.45%)	0.956 (0.542–1.685)	0.02	0.876
	CC	342 (78.26%)	70 (79.55%)	Ref		
	**A**	98 (11.21%)	18 (10.23%)	0.902 (0.530–1.534)	0.14	0.703
	C	776 (88.79%)	158 (89.77%)			
	AA+AC.VS.CC			0.926 (0.526–1.630)	0.07	0.789
	AA.VS.AC+CC			1.426 (0.073–27.846)	0.61	0.436

rs111611328	CC	0 (0%)	0 (0%)	5.023 (0.099–254.873)	NaN	1.00
	GC	3 (0.69%)	2 (2.27%)	3.364 (0.554–20.436)	1.95	0.162
	GG	434 (99.31%)	86 (97.73%)	Ref		
	**C**	3 (0.34%)	2 (1.14%)	3.337 (0.554–20.120)	1.94	0.468
	G	871 (99.66%)	174 (98.86%)			
	CC+GC.VS.GG			3.364 (0.554–20.436)	1.95	0.126
	CC.VS.GC+GG			0.202 (0.004–10.263)	NaN	1.00

rs78726532	GG	3 (0.69%)	0 (0%)	0.671 (0.034–13.130)	0.63	0.426
	AG	94 (21.51%)	16 (18.18%)	0.804 (1.447–1.447)	0.53	0.466
	AA	340 (77.8%)	72 (81.82%)	Ref		
	**G**	100 (11.44%)	16 (9.09%)	0.774 (0.445–1.348)	0.82	0.364
	A	774 (88.56%)	160 (90.91%)			
	GG+AG.VS.AA			0.779 (0.433–1.401)	0.7	0.403
	GG.VS.AG+AA			1.426 (0.073–27.846)	0.61	0.436

**Table 5:** Genotypic distribution of TLR3 gene polymorphisms when chronic HCV patients were compared to patients diagnosed with liver cirrhosis and HCC.

SNPs	Genotype/allele distribution	Chronic HCV	Cirrhosis + HCC	OR (95% C.I.)	*χ* ^2^	*p* value
		*n* = 437	*n* = 126			
rs5743311	AA	0 (0%)	0 (0%)	3.421 (0.068–173.296)	NaN	1.00
	AG	15 (3.43%)	3 (2.38%)	0.686 (0.195–2.409)	0.35	0.554
	GG	422 (96.57%)	123 (97.62%)	Ref		
	**A**	15 (1.72%)	3 (1.19%)	0.690 (0.198–2.402)	0.34	0.779
	G	859 (98.28%)	249 (98.81%)			
	AA+AG.VS.GG			0.686 (0.195–2.409)	0.35	0.554
	AA.VS.AG+GG			0.289 (0.006–14.645)	NaN	1.00

rs5743312	TT	15 (3.43%)	1 (0.79%)	0.217 (0.028–1.662)	2.61	0.106
	CT	110 (25.17%)	29 (23.02%)	0.857 (0.536–1.369)	0.42	0.517
	CC	312 (71.4%)	96 (76.19%)	Ref		
	**T**	140 (16.02%)	31 (12.3%)	0.735 (0.485–1.116)	2.1	0.147
	C	734 (83.98%)	221 (87.7%)			
	TT+CT.VS.CC			0.780 (0.493–1.235)	1.13	0.288
	TT.VS.CT+CC			4.43 (0.581–33.968)	2.47	0.116

rs1879026	TT	13 (2.97%)	2 (1.59%)	0.493 (0.109–2.226)	0.88	0.348
	GT	129 (29.52%)	32 (25.4%)	0.795 (0.506–1.250)	0.99	0.321
	GG	295 (67.51%)	92 (73.02%)	Ref		
	**T**	155 (17.73%)	36 (14.29%)	0.773 (0.522–1.146)	1.65	0.198
	G	719 (82.27%)	216 (85.71%)			
	TT+GT.VS.GG			0.768 (0.494–1.194)	1.38	0.239
	TT.VS.GT+GG			1.901 (0.423–8.537)	0.73	0.394

rs5743313	TT	28 (6.41%)	9 (7.14%)	1.369 (0.611–3.069)	0.59	0.444
	CT	179 (40.96%)	63 (50%)	1.499 (0.992–2.265)	3.72	0.054
	CC	230 (52.63%)	54 (42.86%)	Ref		
	**T**	235 (26.89%)	81 (32.14%)	1.288 (0.951–1.745)	2.68	0.102
	C	639 (73.11%)	171 (67.86%)			
	TT+CT.VS.CC			1.481 (0.993–2.209)	3.74	0.053
	TT.VS.CT+CC			0.890 (0.409–1.939)	0.09	0.769

rs5743314	CC	28 (6.41%)	9 (7.14%)	1.387 (0.619–3.109)	0.64	0.425
	GC	176 (40.27%)	63 (50%)	1.545 (1.022–2.334)	4.29	0.0383
	GG	233 (53.32%)	54 (42.86%)	Ref		
	**C**	232 (26.54%)	81 (32.14%)	1.311 (0.967–1.777)	3.05	0.0805
	G	642 (73.46%)	171 (67.86%)			
	CC+GC.VS.GG			1.523 (1.021–2.271)	4.28	0.0385
	CC.VS.GC+GG			0.890 (0.409–1.939)	0.09	0.769

rs5743315	AA	3 (0.69%)	0 (0%)	0.487 (0.025–9.504)	0.88	0.349
	AC	92 (21.05%)	26 (20.63%)	0.967 (0.593–1.576)	0.02	0.891
	CC	342 (78.26%)	100 (79.37%)	Ref		
	**A**	98 (11.21%)	26 (10.32%)	0.911 (0.577–1.439)	0.16	0.689
	C	776 (88.79%)	226 (89.68%)			
	AA+AC.VS.CC			0.936 (0.575–1.524)	0.07	0.7903
	AA.VS.AC+CC			2.038 (0.105–39.716)	0.87	0.351

rs111611328	CC	0 (0%)	1 (0.79%)	10.555 (0.427–260.710)	3.51	0.061
	GC	3 (0.69%)	2 (1.59%)	2.352 (0.389–14.236)	0.92	0.337
	GG	434 (99.31%)	123 (97.62%)	Ref		
	**C**	3 (0.34%)	4 (1.59%)	4.683 (1.041–21.062)	4.9	0.088
	G	871 (99.66%)	248 (98.41%)			
	CC+GC.VS.GG			3.528 (0.703–17.701)	2.66	0.103
	CC.VS.GC+GG			0.096 (0.004–2.362)	3.47	0.062

rs78726532	GG	3 (0.69%)	0 (0%)	0.475 (0.024–9.263)	0.90	0.343
	AG	94 (21.51%)	24 (19.05%)	0.851 (0.516–1.403)	0.4	0.526
	AA	340 (77.8%)	102 (80.95%)	Ref		
	**G**	100 (11.44%)	24 (9.52%)	0.815 (0.510–1.303)	0.73	0.392
	A	774 (88.56%)	228 (90.48%)			
	GG+AG.VS.AA			0.825 (0.501–1.358)	0.57	0.448
	GG.VS.AG+AA			2.038 (0.105–39.716)	0.87	0.351

**Table 6:** Haplotype frequencies of TLR3 SNPs in chronic HCV-infected patients compared to patients with liver cirrhosis.

Haplotype			Freq.	Liver cirrhosis, chronic HCV ratio counts	Liver cirrhosis, chronic HCV frequencies	Chi square	*p* value
rs1879026	rs5743313	rs5743314					
G	C	G	0.547	90.4 : 85.6, 483.8 : 390.2	0.513, 0.554	0.953	0.329
G	T	C	0.27	59.6 : 116.4, 224.1 : 649.9	0.339, 0.256	5.035	0.0248
T	C	G	0.167	24.6 : 151.4, 151.1 : 722.9	0.140, 0.173	1.144	0.2847

**Table 7:** Haplotype frequencies of TLR3 SNPs in chronic HCV-infected patients compared to patients with cirrhosis + HCC infected patients.

Haplotype			Freq.	Liver cirrhosis + HCC, chronic HCV ratio counts	Liver cirrhosis + HCC, chronic HCV frequencies	Chi Square	*p* value
rs1879026	rs5743313	rs5743314					
G	C	G	0.549	134.5 : 117.5, 483.7 : 390.3	0.534, 0.553	0.311	0.577
G	T	C	0.27	79.5 : 172.5, 224.2 : 649.8	0.315, 0.257	3.452	0.0632
T	C	G	0.166	35.5 : 216.5, 151.2 : 722.8	0.141, 0.173	1.458	0.2273
